# Annual Meeting of the American Medical Association

**Published:** 1880-06-20

**Authors:** 


### *ANNUAL MEETING OF THE AMERICAN MEDICAL ASSOCIATION.*

---

We extract from our exchanges (Boston Med. and Surg. Journal and New York Med. Record) the following brief of the proceedings of the American Medical Association.

As had been anticipated, the thirty-first annual meeting of the American Medical Association was very largely attended, and was one of the most successful and satisfactory sessions of this body which has yet been held. The city of New York, owing to the convenience of access and its numerous attractions, offered unusual inducements for the meetings, which were held in the lecture rooms of the College of Physicians and Surgeons, and in the Young Men's Christian Association rooms, on the southwest corner of Twenty-Third Street and Fourth Avenue, opposite to the college. The course of the proceedings was marked by harmony and dignity; much credit is due to the Committee of arrangements for the admirable manner in which the business and entertainments were provided for. The number of papers that were presented was so great, no less than one hundred and eighty-four having been placed in the hands of the committee, that it was utterly impossible to have them all read at the meeting.

It seems invidious to name only a few of the distinguished men who were present, but in addition to the officers of the meeting—President Prof. Lewis A. Sayre, of New York; Vice-Presidents R. Beverly Cole, of San Francisco, H. O. Marcy, of Massachuset s, F. Peyre Porcher, of South Carolina; Secretary William B. Atkinson, of Philadelphia; Assistant Secretary Walter R. Gillette, of New York—we observed upon the platform Professors H. I. Bowditch, of Boston, H. D. Gross, H. Pancoast, and Robert Bartholow, of Philadelphia, James White, of Buffalo, T. G. Richardson, of New Orleans, John L. Atlee, of Lancaster, Pa, J. Marion Sims, of New York, Dr. Moore, of Rochester, Dr. Lewis, of Philadelphia, Prof. Dunster, of Ann Arbor, Dr. Chadwick, of Boston; and among the distinguished strangers were Jonathan Hutchinson, of London, Dr. Hingston, of Montreal, and Dr. Irenholme, of Canada

The sections were officered as follows:

1. Section of Practice of Med., Materia Med., and Physiology, chairman, J. S. Lynch, M.D., Baltimore; secretary, W. B. Glasgow, M.D., St. Louis.
2. Section of Surgery and Anatomy, chairman, W. T. Briggs, M.D., Nashville, Tenn.; secretary, C. Powell Adams, M.D., Hastings, Minn.
3. Section of Obstetrics and Diseases of Women and Children, Prof. Morse, St. Louis, chairman, (in place of Dr. A. H. Smith, the regular appointee, who was detained by sickness; secretary, Robert Battey, M. D., of Rome, Ga.
4. Section of Medical Jurisprudence, Chemistry, Psychology, State Medicine, and Hygiene, chairman, James F. Hibberd, M.D., Richmond, Ind.; secretary, Thomas F. Wood, M. D., Wilmington, N. C.
5. Section of Ophthalmology, Otology, and Laryngology, chairman, Laurence Turnbull, M.D., of Philadelphia (Dr. Bolling A. Pope, of New Orleans, being absent); secretary, Eugene Smith, M. D., Detroit Michigan.

The Committee of Arrangements made several innovations at this meeting. The first was to encroach upon section three, and set aside a room for the meeting-place of those favoring the organization of a separate section for diseases of children, with the intention of creating a separate section next year. This was done, but of course no further business was transacted before this sub-section. The next innovation was the publication of the programme in daily installments, after the method of the British Medical Association. The amount of business to be transacted made this arrangement necessary, and also justified the third innovation, which was the convention of the sections at two p.m. instead of three o'clock, as generally is the case.

The entertainments provided were very enjoyable; the size and style of the invitation cards and their number have seldom, if ever, been equalled, certainly not excelled.

On Tuesday evening, June 1st, a reception by the members of the medical profession and other citizens of New York was given at the Academy of Music, where Grafulla's orchestra discoursed most eloquent music; the Committee on Entertainment and Invitations being Drs. Chas. Inslee Pardee, Montrose A. Pallen, Robert F. Weir, Jos. C. Hutchinson, Wm. M. Polk, and E. H. Parker.

On Wednesday a complimentary entertainment was given to the members of the American Medical Association and their ladies by Messrs. Reed and Carnrick, Messrs. Scott and Bowne, and the New York Pharmacal Association. On this occasion was presented Othello, with Edwin Booth as Iago. Beautiful silk programmes were distributed.

On the evening of June 3d, Mayor Cooper, Mr. August Belmont, and Drs. Fordyce Barker and T. Gaillard Thomas each gave receptions to the delegates. The last named reception was held at the Academy of Music, and owing to the popularity of the hosts of the occasion it was very largely attended; as, indeed, were those of Messrs. Cooper and Belmont, which were everything that lavish hospitality could make them, to render them enjoyable by the guests.

On Friday afternoon, June 4th, Messrs. Wm. R. Wood & Co. invited the members of the Association upon a steamboat excursion around New York harbor, stopping at Brighton beach.

The General Session was called to order on June 1st., 11 o'clock by Pres. Lewis A. Sayre, of New York. Dr. Gaillard Thomas made the address of welcome, which was followed by an able address of President Sayre on the subject of "Improvement of Medical and Surgical Science in America." The officers chosen for the next year are as fol ows:

For President—John T. Hodgen, M.D., of St. Louis, Mo.

For Vice Presidents—1st, W. H. Anderson, M.D., of Mobile, Ala. 2d, Levi G. Hill, of New Hampshire. 3d, Henry T. Holton, of Vermont. 4th, H. C irpenter, of Oregon.

For Permanent Secretary—W. B. Atkinson, M.D., of Philadelphia, Pa.

For Treasurer—R. Dunglison, M.D., of Philadelphia, Pa.

For Librarian—Wm. Lee, M.D., Washington, D.C.

For Chairman of the Section on Practice of Medicine, Materia Medica, and Physiology—Dr. Charles Denison, of Colorado.

For Secretary—Dr. T. Ashby, of Maryland.

For Chairman of the Section on Surgery and Anatomy—Dr. H. McGuire, of Richmond, Va.

For Secretary—Dr. D. A Eve, of Tennessee.

For Chairman of the Section on Obstetrics and Diseases of Women—Dr. James R. Chadwick, of Boston, Mass.

For Secretary—Dr. J. Taber Johnson, of Washington, D.C.

For Chairman of the Section on Medical Jurisprudence and State Medicine—Dr. J. Reeves, of Wisconsin.

For Secretary—Dr. R. G. Young, of Arkansas.

For Chairman of the Section on Ophthalmology, Otology and Laryngology—Dr. D. S. Reynolds, of Kentucky.

For Secretary—Dr. S. M. Burnett, of Washington, D. C.

For Members of the Judicial Council, to fill vacancies—Drs. J. K. Bartlett, of Wisconsin; F. Staples, of Minnesota; D. R. Wallace, of

Texas; J. S. Billings, of U. S. Army; J. H. Warren, of Massachusetts; and A. T. Woodward, of Vermont.

The Committee recommended that the next meeting of the Association be held in the City of Richmond, Va., on the first Tuesday in May, 1881.

As Chairman of Committee of Arrangements—Dr. F. D. Cunningham, of Richmond, Va.

For Assistant Secretary—Dr. J. G. Cabell, of Virginia.

For Committee of Arrangements—Drs. Cunningham, McGuire and Cullen, of Richmond, Va., with power to select others to make a Committee of seven.

For Committee of Publication—The same as last year. Drs. W. B. Atkinson, T. M. Drysdale, William Lee, R. J. Dunglison, Albert Fricke, S. D. Gross and Casper Wistar, all of Philadelphia.

For Chairman of the Section on Practice of Medicine—Dr. Wm. Pepper, of Pa.

For Chairman of the Section on Diseases of Children—Dr. A. Jacobi, of New York.

For Secretary—Dr. W. H. Bradford, of Boston.

Dr. Hibberd, Chairman of the Section on Medical Jurisprudence and State Medicine, offered the following resolutions as sent up from the Section:

First—Endorsing the National Board of Health.

Second—That the Association recommend medical schools to establish a Chair of State Medicine as a part of the regular curriculum.

Third—That the name of the Section hereafter shall be, "Section on State Medicine."

Fourth—That the Committee on Prize Essays shall be Drs. S. E. Chaille, of La., J. L. Cabell, of Va. and A. N. Bell, of N. Y.

The President introduced Dr. John T. Hodgen, of St. Louis, the President-elect, who responded appropriately to the call of the Association.

The Association was then declared adjourned to meet in the City of Richmond, Va., on the first Tuesday in May, 1881.

